# Changes in Muscle Strength in U19 Soccer Players During an Annual Training Cycle

**DOI:** 10.2478/hukin-2014-0072

**Published:** 2014-10-10

**Authors:** Michal Lehnert, Zuzana Xaverová, Mark De Ste Croix

**Affiliations:** 1 Faculty of Physical Culture, Palacký University in Olomouc, Czech Republic.; 2 School of Sport and Exercise, University of Gloucestershire, Gloucester, United Kingdom.

**Keywords:** adolescents, H/Q ratio, isokinetic, torque, training

## Abstract

The aim of the study was to investigate the seasonal variation in isokinetic strength of the knee flexors and extensors, and conventional (H/Q_CONV_) and functional (H/Q_FUNC_) hamstring to quadriceps strength ratios in highly trained adolescent soccer players. The players (n=11; age 17.8±0.3) were measured at the end of the competitive season (autumn), at the beginning and the end of pre-season (winter) and during the sixth week of a new competitive season. Isokinetic peak torque (concentric and eccentric) was measured at 60°·s-1 in a sitting position with the hip flexed at 100°. The testing range of motion was set from 10 – 90° of knee flexion. The players performed a set of five maximum repetitions for both the dominant and non-dominant leg. Statistically significant differences (p<0.001) between the four seasonal measurements were noted for peak torque of the dominant leg knee flexors in concentric muscle action only. A post hoc analysis revealed a statistically significant increase in peak torque from the 1st to the 4th measurement (p<0.001; d=0.692) and from the 2nd to the 4th (p<0.01; d=0.564). The differences in the changes of peak torque of the knee flexors and extensors depending on type of muscle action and tendencies found in the H/Q ratios throughout the annual training cycle indicate that strength assessment of the knee flexors and extensors and their balance throughout the annual training cycle could be beneficial for elite male adolescent soccer players both in terms of performance and risk of injury.

## Introduction

In contemporary top sport, a high level of physical fitness is one of the key preconditions for success. In soccer, the main components of soccer-specific fitness include acceleration, anaerobic repeated sprint ability and explosive power of the lower extremities. These components are, in particular, associated with the production of dynamic force during running, turns, kicking, jumping, tackling and maintaining balance (Psotta et al., 2011; Stølen et al., 2005). The knee extensors are the prime movers involved in running, jumping and kicking the ball, whereas the knee flexors are involved in running where they influence stride length and stabilize the knee joint in changes of direction, acceleration and deceleration, and during landing (Cerrah et al., 2011; Stølen et al., 2005). Strength of the knee flexors and extensors and their ratios has also been identified as an important parameter in prevention of injury of the knee and hamstrings (Huges and Watkins, 2006; Proske et al., 2004). A frequent and often traumatic injury of the knee involves the anterior cruciate ligament (ACL) (Walden et al., 2011) and in soccer, the incidence of this type of injury appears to increase throughout adolescents (Lohmander et al., 2007).

Examining strength of the knee flexors and extensors and their reciprocal muscle group ratios using isokinetic dynamometry provides information on knee function, risk of injury, and more importantly, knee joint stability (Aagaard et al., 1998). Although there is no absolute consensus about a normative value, it is generally accepted that if the conventional H/Q ratio (H/Q_CONV_), i.e. the concentric hamstring peak torque (PT) divided by the concentric quadriceps PT does not exceed 0.6 at a speed of 60°·s-1, the probability of hamstring and ACL injury increases (Heiser et al., 1984).

While H/Q_CONV_ indicates muscle imbalance, functional H/Q ratio (H/Q_FUNC_), i.e. eccentric hamstring PT divided by concentric quadriceps PT, expresses the ability of the knee flexors to decelerate leg extension performed by the quadriceps (Coombs and Garbutt, 2002). An optimum value of the H/Q_FUNC_ ratio is influenced by joint angle, individual differences in the proportion of muscle fibres and the specific type of sport. It has been suggested that at an angular velocity of 60°·s-1 a ratio of 1.0 (determined using PT values obtained in the mid-range of knee joint movement) indicates adequate joint stability (Coombs and Garbutt, 2002). Thus, values for the H/Q_FUNC_ ranging from 0.7–1.0 have been suggested to indicate insufficient dynamic stabilization of the knee (Aagaard et al., 1995; Aagaard et al., 1998). In recent research studies a greater increase in the strength of the quadriceps compared with hamstrings has been found in adolescent soccer players, what has been attributed to traditional forms of soccer training (Iga et al., 2010; Lehance et al., 2009). As a result, a reduced H/Q_FUNC_ ratio caused by traditional soccer training may place this population at a greater relative risk of knee injury.

An important aim of the training process during annual training cycles is to increase or to maintain muscle strength and power at a consistent level (Fleck and Kraemer, 2004). However, during the post-season and in-season the loss of adaptations is a serious problem (Mujika and Padilla, 2000b) which may negatively affect both game performance and risk of injury in players. It is surprising therefore it appears that isokinetic strength of the lower extremities has been measured during repeated periods of a season in only two studies in adult professional soccer players (Malliou et al., 2003; Eniseler et al., 2012). In a study by Malliou et al. (2003), concentric PT of the knee extensors at a speed of 60°·s-1 and 180°·s-1 was measured at the end of the competitive season, after post-season and after pre-season. The results indicated no significant change in average PT values during the observed periods. In a study by Eniseler et al. (2012), PT of the knee flexors and extensors was determined at three different velocities and H/Q_CONV_ ratios calculated over the course of 24 weeks (pre-season and competitive season). The results showed no significant changes at a velocity of 60°·s-1. However, PT improved significantly only at the highest movement velocity (500°·s-1) and the H/Q_CONV_ ratio increased significantly at a velocity of 500°·s-1 for both DL and NL and at a velocity of 300°·s-1 for DL. The results of the aforementioned studies in adult players indicate that there is a minimum change in isokinetic strength parameters measured at a low velocity between different phases of a soccer training cycle. However, these findings cannot be applied to teenage boys because of developmental changes in strength during normal growth and maturation (De Ste Croix et al., 2003).

To our knowledge no study has investigated seasonal variation in PT and in H/Q ratios during the various training phases in youth soccer players. It still needs to be identified how muscle strength of the knee flexors and extensors and their ratios are altered throughout the annual training cycle in elite youth male soccer players. Therefore, the aim of the study was to investigate the seasonal variation in isokinetic strength of the knee flexors and extensors and H/Q ratios in highly trained adolescent soccer players.

## Material and Methods

### Participants

The complete testing was carried out on eleven soccer players of a U19 men’s team (age 17.8±0.3 years) who played the first division U19 (Table 1). The reason for the low number of subjects included in the study was that players with acute medical problems and with a history of knee-related injury were excluded from the research likewise any player who did not complete all measurements. All players self-reported the right leg as their dominant leg. Leg dominance was verified (preferred kicking leg) before testing. The study was approved by the ethics committee of the Faculty of Physical Culture, Palacký University in Olomouc and conformed to the Declaration of Helsinki regarding the use of human subjects. All tested players were fully informed about the aim of the study and the testing procedures employed in the study. A written informed consent to the testing procedures and the use of the data for further research was obtained. The day before testing, the evaluated players were not exposed to intensive training. All the players were experienced in iskokinetic testing.

### Procedures

Bilateral isokinetic strength of the knee flexors and extensors was measured using an isokinetic dynamometer IsoMed 2000 (D. & R. Ferstl GmbH, Hemau, Germany). Prior to testing the players completed non-specific warm-up exercises, which included cycling on a stationary bicycle ergometer for 6 minutes at a self-regulated low to moderate intensity, 5 minutes of dynamic stretching exercises which targeted the main muscle groups involved during testing and ten vertical jumps with progressive jump height. The warm-up routine was performed under the supervision of the researcher. The players were tested in a sitting position with a hip angle of 100°. For fixation of the pelvis and the thigh of the tested leg, fixed straps were used; the shoulders were fixed by shoulder pads in the ventral-dorsal and cranialcaudal direction. The axis of rotation of the dynamometer was aligned with the axis of rotation of the knee (lateral femoral epicondyle). The arm of the dynamometer lever was fixed to the distal part of the shin and the lower edge of the shin pad was placed 2.5 cm over the medial apex malleolus. Individual seat settings were stored on the computer before measuring the right leg and were automatically activated in the process of measuring the left leg and follow-up testing respectively. At the beginning of the follow-up testing, individual settings were rechecked and adjusted if necessary. The participants were instructed to hold the handgrips located at the side of the seat during all testing efforts. An angular velocity of 60°·s^−1^ was used for the measurement. Static gravitational correction was applied according to the manufacturer’s procedures. The testing range of motion was 80° and was set from 10 – 90° of knee flexion (with 0° = full voluntary extension). The testing protocol consisted of two contraction sets (warm-up and testing) of concentric and eccentric muscle actions. The concentric muscle action preceded the eccentric muscle action and there was a 1-minute rest interval between the set. In the first warm-up set the players performed five concentric/concentric reciprocal actions (with flexion movements performed first) with a progressive rise in the muscle action until a maximum action was performed. After a 1-minute rest the players performed a set of six maximum repetitions. The rest time between the measurement of the dominant leg (DL) and nondominant leg (NL) was 3 minutes, and the DL was measured first. During the testing procedure the players were provided with concurrent visual feedback in the form of an isokinetic strength curve displayed on the dynamometer monitor. Verbal encouragement was not provided. Absolute peak torque, H/Q_CONV_ and H/Q_FUNC_ were determined. The H/Q ratios were determined using PT value for concentric and an eccentric action.

Four measurement sessions were undertaken during the different phases of the training process. The 1^st^ measurement was undertaken at the end of the autumn part of the competitive season, the 2^nd^ measurement at the beginning of the pre-season after a three-week period without training, the 3^rd^ at the end of the winter pre-season (after 8 weeks) and the 4^th^ in the sixth week of the spring competitive season (after 6 weeks).

### Training programme

The training content including matches and recovery periods was recorded, and the values of approximate duration of the respective types of activities used with regard to the main training effects were calculated based on the training programme (including training adaptations made by the coach) (Table 2).

#### Statistical analysis

The mean, median and standard deviation values were calculated for PT and H/Q ratios. A one-way ANOVA was used to determine the significance of PT differences between the measurements (p<0.05). The effect size was assessed by the “Partial Eta squared” coefficient (η^2^). The statistical significance of the differences between the results of particular measurements was verified by a post hoc Scheffe’s test. The effect size was assessed by the Cohen’s coefficient (d<0.2–0.5 small difference; d<0.5–0.8 medium difference; d<0.8 large difference) (Cohen, 1988). Statistical analysis was performed using the data analysis software system Statistica, version 10 (StatSoft, Inc., Tulsa, USA).

## Results

### Seasonal variability in isokinetic muscle strength of the knee flexors and extensors

**Figure 1:**
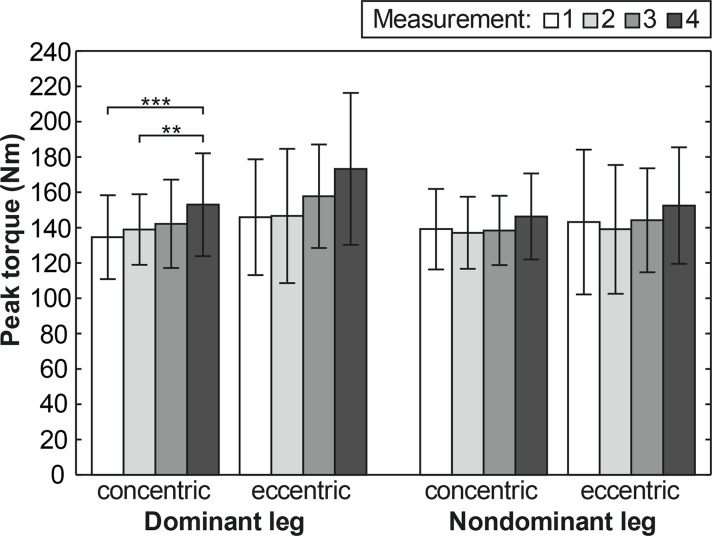
Peak torque (mean and standard deviation) for flexors at 60°·s^−1^ for individual measurements (^**^p<0.01, ^***^p<0.001)

**Figure 2:**
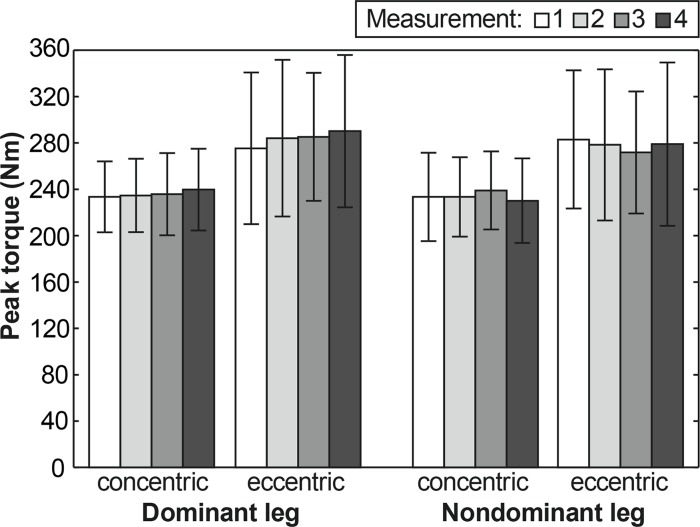
Peak torque (mean and standard deviation) for extensors at 60°·s^−1^ for individual measurements

PT values for flexors and extensors of DL and NL for all four measurements are reported in Table 3. A Friedman ANOVA showed a statistically significant difference in PT between the three seasonal measurements for DL knee flexors in concentric muscle actions only (p<0.001; η^2^=0.433) while measurement time was not a statistically significant factor for PT of the knee extensors of both lower extremities. In the case of DL knee flexors in concentric muscle action, a post hoc analysis revealed a statistically significant increase in PT from the 1^st^ to the 4^th^ measurement (p<0.001; d=0.692) and from the 2^nd^ to the 4^th^ measurement (p<0.01; d=0.564). However, an increase in PT from the 3^rd^ to the 4^th^ measurement was on the border of statistical significance (p<0.080). For flexors PT there was a significant increase in concentric muscle action for DL only in the monitored training phase (Figure 1). For extensors all the changes for both concentric and eccentric actions were non-significant (Figure 2).

### Seasonal variability in H/Q ratios

**Figure 3:**
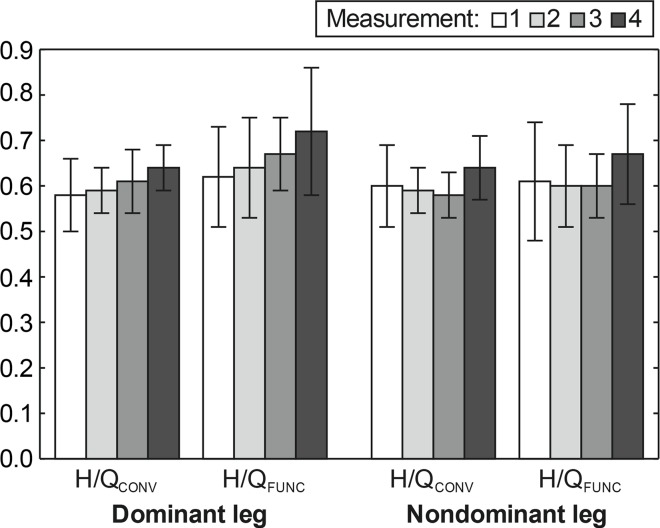
H/Q_CONV_ and H/Q_FUNC_ (mean and standard deviation) for individual measurements

The changes in mean values for H/Q_CONV_ ratio and H/Q_FUNC_ ratio of the DL and NL can be seen in Figure 3. A significant effect of time was not observed for both DL and NL. In the 1^st^, 2^nd^ and 3^rd^ measurement the average values of H/Q_CONV_ ratio for DL and NL did not exceed a normative value of 0.6 and in the 4^th^ measurement (spring competitive season) the average values were just above the normative. During the monitored period, the mean values for H/Q_FUNC_ ratio did not significantly increase for DL, whereas for NL the values plateaued and increased in the fourth measurement (0.61; 0.60; 0.60; 0.7). However, the values were in the range of 0.6–0.7 except the fourth measurement for DL.

## Discussion

### Isokinetic peak torque

The results of the study indicated that PT in the knee flexors and extensors at an angle velocity of 60°·s^−1^ after the autumn competitive season, at the beginning and at the end of the off-season and during the spring competitive season changed significantly only for DL flexors during concentric actions (p<0.001). Nevertheless, we observed a significant increasing trend of the average values of flexor PT of DL also in the eccentric mode; in the last measurement the PT values increased by 18.6% compared with the first measurement. The observed increase could be considered beneficial in terms of the players’ physical status, however, a smaller increase (6.7%) was found for NL. With respect to identical load of DL and NL during strength training the increase could be explained by differences in neural adaptations as a result of different engagement of the lower extremities in high-intensity movements during specific soccer training that increased prior to and during the competitive season. However, it could be anticipated that these neural adaptations would have an effect particularly during isokinetic testing at higher angular velocities (Eniseler et al., 2012).

The data show that after a three-week period without training the strength of the knee flexors and extensors did not decrease. Although there might be a decrease in physiological adaptations as a result of reduced or interrupted adaptation stimuli (Mujika and Padilla, 2000a; Mujika and Padilla, 2000b), this finding is not surprising because the players were not subjected to organised strength training or organised match play for only three weeks, which is a short period for de-adaptation to take place (Mujika and Padilla, 2000b). It would be interesting in future studies to investigate if neuromuscular de-training occurs during this period which might predispose youth soccer players to greater injury risk at the start of pre-season training.

After the pre-season the PT of the knee flexors and extensors of both lower extremities did not change significantly. The cause of the plateauing in PT could have been a low frequency (once a week) of resistance training (Fleck and Kraemer, 2004; Hoff, 2005). Another explanation could also be the concurrent application of strength, speed and endurance training. Although the opinions on training programmes of such character are not completely uniform (Fleck and Kraemer, 2004) it is suggested that the effectiveness of strength training may be lowered. The results of the current study are comparable with the results of a study by Botek et al. (2010) focusing on players of the same U19 category. Also in this sample the PT value measured in the concentric mode at an angular velocity of 60°·s^−1^ after the pre-season increased but not significantly only in the DL flexors (PT_1_=165.4±1 Nm; PT_2_=169.3±3 Nm). Also Malliou et al. (2003) reported a plateauing and even a small decrease in extensor PT at velocities of 60°·s^−1^ and 180°·s^−1^ in senior soccer players after the pre-season.

In the fourth measurement (the sixth week of the spring competitive season) the current group achieved the highest average values of PT in the flexors for both concentric and eccentric actions of DL and NL. Although the increase was statistically significant only for DL in the concentric mode, in terms of game performance and risk injury the difference in the eccentric mode between the first and fourth measurement (18.7%) and between the second and fourth measurement (18.1%) must also be considered. The increased knee flexor PT observed in the fourth measurement in both lower extremities may possibly be attributed to a positive effect of core training and resistance training (incorporated both complex exercises for the lower extremity and exercise for hamstring strengthening) applied once a week and soccer-specific exercises with fast muscle actions. Another possible explanation of the flexor PT increase at the beginning of the competitive season may be a decrease in the training load during the competitive season and the disappearance of any residual fatigue, which may have reduced strength production immediately after the pre-season. The results of a previous study (Sangnier and Tourny-Chollet, 2007) indicated that acute effects of fatigue were significantly greater in the hamstrings compared with the quadriceps in professional adult male soccer players.

The findings of the current study differ from the results of a study by Eniseler et al. (2012) in which PT of the knee flexors and extensors in adult players did not change significantly at velocities of 60°·s^−1^ and 300°·s^−1^ during the pre-season and competitive season. However, an increase at the highest velocity of 500°·s^−1^ was significant. Changes of PT throughout the season could be also influenced by the entry level of strength of the players. Comparison of our group with groups of players of a similar age irrespective of the training phase indicates that the players in our group achieved similar average PT values (Lehance et al., 2009; Maly et al., 2010) or higher PT values (Forbes et al., 2009).

### Isokinetic H/Q ratios

Muscle strength of the knee flexors and extensors should be well balanced in players throughout the season to reduce the risk of lower limb injury. The main finding was that during the monitored period no statistically significant changes in the H/Q_CONV_ and H/Q_FUNC_ ratios in the lower extremities calculated from PT at an angular velocity of 60°·s^−1^ were observed in our sample.

The values of the H/Q_CONV_ ratio were just below or on the limit of the proposed normative value of 0.6; this value was exceeded only in the third and fourth measurement (DL), and in the fourth measurement (NL) (Figure 3). Despite the fact that in the fourth measurement there was a significant increase in hamstring PT in concentric muscle action, the ratio of hamstring and quadriceps strength in both lower extremities in this period just slightly exceeded the value of 0.6.

The observed changes in the H/Q_CONV_ ratio correspond with the results of a study by Eniseler et al. (2012). However, this study was performed on senior Turkish professional soccer players during 24 weeks of the pre-season and in-season. The measurement was carried out at three velocities. At a velocity of 60°·s^−1^ the authors observed changes in the H/Q_CONV_ ratio from 55% to 63% (DL) and from 56% to 61% (NL), which were not statistically significant. However, the values of the H/Q_CONV_ ratio in our players irrespective of the training phase are comparable with the results of previous studies of groups of a similar age. The players in the current study achieved better results compared with players of a similar age in a study by Maly et al. (2010), and similar results were also achieved by U17 players in a study by Lehance et al. (2009) and U18 players in a study by Forbes et al. (2009).

In terms of the H/Q_FUNC_ ratio, during the monitored period we observed an increasing trend in our sample (DL from 0.62 to 0.72; NL from 0.60 to 0.67), which indicates a decreased degree of strength imbalance in the knee flexors and extensors between the third (end of pre-season) and fourth measurement (spring competitive season). In particular, this trend reflects an increase in flexor PT during eccentric and also concentric muscle action in the fourth measurement. In spite of that it is obvious that during the monitored period the average value of the sample did not come close to 1.0, which is a value indicating a balance between the strength of the knee flexors and extensors, i.e. their optimum capability of dynamic knee stabilization (Coombs and Garbutt, 2002).

Also a comparison of the results of our group irrespective of the training phase with the values observed in players of a similar age in other studies reveals that the values of the H/Q_FUNC_ ratio are low. In a study aimed at U12 to U18 players, Forbes et al. (2009) measured values of 0.85–1.10. At the same time the authors note that the worst results were achieved by the U18 category. Similar results (0.76–1.29) were also reported by Kellis et al. (2001) in a study of 10 to 17-year-old players, where in the oldest U17 category the following average values were observed (DL 0.79±0.17; NL 0.80±0.21). The values of the H/Q_FUNC_ ratio found suggest that the players of our group were quadriceps dominant (Iga et al., 2010). However the positive changes of this ratio from the 3^rd^ to the 4^th^ measurement show that they were responsive to the conditioning which included hamstring exercises (both concentric and eccentric) and that their enhancement could further reduce the risk of injury in the players.

One of the limitations of the study is PT measurement at a single angular velocity. For a more detailed assessment of the capability of dynamic knee stabilization in soccer players it would be appropriate to measure PT also at a higher angular velocity (Aagaard et al., 1995). Another limitation is a low number of players who completed the whole measurement and were involved in the study.

## Conclusions

In conclusion, the results of the current study in elite youth soccer players indicate that in the monitored training phases of the annual training cycle, the isokinetic strength of the knee flexors and extensors varied with significant differences in PT for DL knee flexors during concentric actions only. Although the assessment of strength imbalance of the knee flexors and extensors using H/Q_CONV_ and H/Q_FUNC_ did not reveal any significant changes in the selected periods of the annual training cycle for both DL and NL, the observed tendencies, especially in case of H/Q_FUNC_, indicate that muscle strength imbalance of the knee flexors and extensors changed throughout the annual training cycle. Therefore, a repeated assessment of muscle strength of the knee flexors and extensors and their balance throughout the annual training cycle could be beneficial for elite male adolescent soccer players both from the point of view of performance and injury risk. For this purpose we recommend to use isokinetic dynamometry focused on both concentric and eccentric muscle actions.

## Figures and Tables

**Table 1:** Stature (cm) and body mass (kg) of participants (n=11)

Variable	*n*	*M*	*Mdn*	*Min*	*Max*	*SD*
Stature	11	180.77	181.50	174.50	187.00	4.46
Body mass 1	11	72.22	71.93	63.84	84.71	7.17
Body mass 2	11	71.75	70.64	63.52	83.22	7.14
Body mass 3	11	71.78	71.84	63.82	85.12	7.38
Body mass 4	11	72.62	73.02	63.56	86.07	7.47

1, 2, 3, 4 – order of testing; M – mean; Mdn – median; Min – minimum; Max – maximum; SD – standard deviation

**Table 2:** Training load during particular months of the observed period

Type of training	January (min)	February (min)	March (min)	April (min)
***Physical Fitness Training***				
Strength and Power	400^*^	165^**^	100^**^	200^**^
Anaerobic Training	120	65	150	105
Aerobic Training	670	210	250	165
***Skill-Oriented Training***				
Technical-Tactical Training	165	325	440	450
Game-Like Training	275	300	470	375
***Matches***	360	540	450	360
***Swimming***	0	90	90	0
***Passive Regeneration***	180	180	135	90
***TOTAL***	2170	1875	2085	1745

^*^ core training; resistance training (once a week);

^**^ core training + plyometric training (once a week) or resistance training (back squats, leg extensions, dumbbell lunges, lying leg curls) – alternatively once a wee

**Table 3:** Peak torque (Nm) of the knee flexors and extensors of the dominant and non-dominant lower extremity – basic statistical characteristics (n=11)

*Muscle group*	*Measurement*	*Leg*	*Concentric action*		*Eccentric action*	

			*M ± SD*	*Mdn*	*M ± SD*	*Mdn*
Flexors	1	DL	134.64 ± 23.71	132	145.91 ± 32.81	145
		NL	139.18 ± 22.08	136	143.18 ± 41.03	126
	2	DL	138.91 ± 19.99	133	146.70 ± 38.02	143
		NL	137.09 ± 20.37	130	139.10 ± 36.48	123
	3	DL	142.18 ± 24.98	129	157.82 ± 29.30	157
		NL	138.45 ± 19.62	133	144.18 ± 29.44	138
	4	DL	153.00 ± 29.06	142	173.27 ± 43.03	165
		NL	146.36 ± 24.40	145	152.55 ± 32.97	144
Extensors	1	DL	233.55 ± 30.62	220	275.36 ± 65.43	262
		NL	233.45 ± 38.15	219	283.00 ± 59.57	259
	2	DL	234.73 ± 31.64	226	284.20 ± 67.59	276
		NL	233.45 ± 34.26	232	278.40 ± 65.22	256
	3	DL	235.82 ± 35.48	244	285.27 ± 55.24	277
		NL	239.00 ± 33.67	237	271.82 ± 52.61	255
	4	DL	239.82 ± 35.26	238	290.27 ± 65.69	274
		NL	230.18 ± 36.51	238	279.09 ± 70.48	259

Con – concentric action; Ecc – eccentric action; F – flexion; E – extension; No – number of measurement; DL – dominant leg; NL – non-dominant leg; M – mean; Mdn – median; SD – standard deviation

## References

[b1-jhk-42-175] Aagaard P, Simonsen EB, Magnusson SP, Larsson B, Dyhre-Poulsen P (1998). A new concept for isokinetic hamstring: quadriceps muscle strength ratio. Am J Sports Med.

[b2-jhk-42-175] Aagaard P, Simonsen EB, Trolle M, Bangsbo J, Klausen K (1995). Isokinetic hamstring/quadriceps strength ratio: influence from joint angular velocity, gravity correction and contraction mode. Acta Physiol Scand.

[b3-jhk-42-175] Botek Z, Gaba A, Lehnert M, Pridalova M, Varekova R, Botek M, Langer F (2010). Condition and Body Constitution of Soccer players category U19 before and after Completing the preparatory period. Acta Univ Palack Olomuc Gymn.

[b4-jhk-42-175] Cerrah AO, Gungor EO, Soylu AR, Ertan H, Lees A, Bayrak C (2011). Muscular activation patterns during the soccer in-step kick. Isokinet Exerc Sci.

[b5-jhk-42-175] Cohen J (1998). Statistical power analysis for the behavioral sciences.

[b6-jhk-42-175] Coombs R, Garbutt G (2002). Developments in the use of the hamstring/quadriceps ratio for the assessment of muscle balance. J Sport Sci Med.

[b7-jhk-42-175] Cotte T, Chatard JC (2011). Isokinetic strength and sprint times in English premier league football players. Biol Sport.

[b8-jhk-42-175] Croisier JL, Ganteaume S, Binet J, Genty M, Ferret JM (2008). Strength imbalances and prevention of hamstring injury in professional soccer players: A prospective study. Am J Sport Med.

[b9-jhk-42-175] De Ste Croix MB, Deighan MA, Armstrong N (2003). Assessment and interpretation of isokinetic muscle strength during growth and maturation. Sports Med.

[b10-jhk-42-175] Eniseler N, Şahan Ç, Vurgun H, Mavi HF (2012). Isokinetic strength responses to season-long training and competition in turkish elite soccer players. J Hum Kinet.

[b11-jhk-42-175] Fleck SJ, Kraemer WJ (1987). Designing resistance training programs.

[b12-jhk-42-175] Forbes HA, Sutcliffe S, Lovell A, McNaughton LR, Siegler JC (2009). Isokinetic thigh muscle ratio in youth football: effect of age and dominance. Int J Sports Med.

[b13-jhk-42-175] Gerodimos V, Mandou V, Zafeiridis A, Ioakimidis P, Stavropoulos N, Kellis S (2003). Isokinetic peak torque and hamstring/quadriceps ratios in young basketball players. J Sport Med Phys Fit.

[b14-jhk-42-175] Heiser TM, Weber J, Sullivan G, Clare P, Jacobs RR (1984). Prophylaxis and management of hamstring muscle injuries in intercollegiate football players. Am J Sports Med.

[b15-jhk-42-175] Hoff J (2005). Training and testing physical capacities for elite soccer players. J Sport Sci.

[b16-jhk-42-175] Hughes G, Watkins J (2006). A risk-factor model for anterior cruciate ligament injury. Sports Med.

[b17-jhk-42-175] Iga J, George K, Lees A, Reilly T (2009). Cross-sectional investigation of indices of isokinetic leg strength in youth soccer players and untrained individuals. Scand J Med Sci Spor.

[b18-jhk-42-175] Kellis S, Gerodimos V, Kellis E, Manou V (2001). Bilateral isokinetic concentric and eccentric strength profile of the knee extensors and flexors in young soccer players. Isokinet Exerc Sci.

[b19-jhk-42-175] Lehance C, Binet J, Bury T, Croisier JL (2009). Muscular strength, functional performances and injury risk in professional and junior elite soccer players. Scand J Med Sci Spor.

[b20-jhk-42-175] Lohmander LS, Englund PM, Dahl LL, Roos EM (2007). The long term consequence of anterior cruciate ligament and meniscus injuries: osteoarthritis. Am J Sports Med.

[b21-jhk-42-175] Malliou P, Ispirlidis I, Beneka A, Taxildaris K, Godolias G (2003). Vertical jump and knee extensors isokinetic performance in professional soccer players related to the phase of the training period. Isokinet Exerc Sci.

[b22-jhk-42-175] Maly T, Zahalka F, Mala L (2010). Isokinetic strength, ipsilateral and bilateral ratio of peak muscle torque in knee flexors and extensors in elite young soccer players. Acta Kin.

[b23-jhk-42-175] Mujika I, Padilla S (2000a). Detraining: Loss of training-induced physiological and performance adaptations. Part I. Sports Med.

[b24-jhk-42-175] Mujika I, Padilla S (2000b). Detraining: Loss of training-induced physiological and performance adaptations. Part II. Sports Med.

[b25-jhk-42-175] Psotta R, Bunc V, Hendl J, Tenney D, Heller J (2011). Is repeated sprint ability of soccer players predictable from field-based or laboratory physiological tests?. J Sports Med Phys Fitness.

[b26-jhk-42-175] Proske U, Morgan DL, Brockett CL, Percival P (2004). Identifying athlets at risk of hamstrings strains and how to protect them. Clin Exp Pharmacol P.

[b27-jhk-42-175] Sangnier S, Tourny-Chollet C (2007). Comparison of the decrease in strength between hamstring and quadriceps during isokinetic fatigue testing in semiprofessional soccer players. Int J Sports Med.

[b28-jhk-42-175] Stølen T, Chamari K, Castagna C, Wisloff U (2005). Physiology of soccer. Sports Med.

[b29-jhk-42-175] Tourny-Chollet C, Leroy D, Léger H, Beuret-Blanquart F (2000). Isokinetic knee muscle strength of soccer players according to their position. Isokinet Exerc Sci.

[b30-jhk-42-175] Walden M, Hagglund M, Werner J, Ekstrand J (2011). The epidemiology of anterior cruciate ligament injury in football (soccer): A review of the literature from a gender-related perspective. Knee Surg Sport Tr A.

